# Review of Chinese medicine intervention in PI3K/AKT pathway to regulate fibrosis

**DOI:** 10.1097/MD.0000000000042957

**Published:** 2025-07-11

**Authors:** Shu-ping Huang, Ze-chao Zhang, Yu Chen, Chang-jie Shang, Min Zhu, Wei-hong Li

**Affiliations:** a Department of Traditional Chinese Medicine Surgery, Ruikang Hospital Affiliated to Guangxi University of Chinese Medicine, Nanning, China; b Department of Yao College of Medicine and Graduate School, Guangxi University of Chinese Medicine, Nanning, China.

**Keywords:** fibrosis, pathway, PI3K/AKT, review, traditional Chinese medicine

## Abstract

The phosphatidylinositol-3-kinase (PI3K)/protein kinase B (AKT) signaling pathway plays a crucial role in the regulation of fibrosis, a pathological process characterized by excessive deposition of extracellular matrix components leading to tissue scarring and dysfunction. Traditional Chinese medicine (TCM) has been increasingly recognized for its potential therapeutic effects in fibrosis by targeting various signaling pathways, including the PI3K/AKT pathway. This review aims to summarize the recent advancements in TCM interventions targeting the PI3K/AKT signaling pathway for the regulation of fibrotic diseases. Recent studies have explored the potential of TCM in the prevention and treatment of fibrotic diseases, particularly through the modulation of the PI3K/AKT signaling pathway. To gather information on TCM and the PI3K/AKT pathway, an extensive search was conducted across various scientific databases, such as Google Scholar, Web of Science, Scifinder, Baidu Scholar and PubMed. TCM has demonstrated unique potential in managing fibrotic diseases through the modulation of the PI3K/AKT signaling pathway. About 37 types of TCM monomers, 68 species of extracts and related compounds, and 50 types of TCM formulas. It discusses their treatment effects on fibrosis in various organs by regulating the PI3K/AKT pathway. The current advancements in TCM interventions targeting the PI3K/AKT pathway offer novel perspectives and strategies for the management of fibrotic diseases. TCM has shown positive effects in treating fibrotic diseases.

## 1. Introduction

Fibrosis is a pathological process that is often induced by cell injury and inflammatory response. It is characterized by excessive deposition of extracellular matrix (ECM) and involved in a variety of diseases, such as hepatic fibrosis, kidney fibrosis, and pulmonary fibrosis. The common feature of these diseases is the replacement of normal tissue architecture by dysplastic fibrous tissue, leading to loss of organ function. Fibrosis is a form of wound repair. When this repair is out of control (pathological repair), fibrous connective tissue increases and parenchymal cells decrease, which causes organ structural damage and dysfunction until organ failure, and the prognosis is poor.^[1]^ Epidemiological studies have shown that fibrotic diseases are globally distributed and the incidence is increasing year by year, which has become a serious global health problem and has a significant impact on the quality of life and survival rate of patients.^[2]^ Phosphatidylinositol-3-kinase (PI3K)/protein kinase B (AKT) signaling pathway is the main effector molecule of TGF-β pathway. They play important roles in physiological processes such as cell proliferation, differentiation, migration and apoptosis.^[3]^ PI3K is a group of lipid kinases associated with the plasma membrane, which can be divided into 3 classes: class I, class II, and class III.^[4]^ The class I PI3K subfamily includes 4 members in vertebrates, class I PI3Ks function as heterodimers, “It consists of one of 4 catalytic p110 subunits (p110α, β, δ, or γ) and a regulatory subunit (p85α [or its splice variants p55α and p50α], p85β, p55γ, p101, or p84).” Class II PI3K (PI3KC2) subfamily has 3 members in vertebrates, including PI3Kc2α, PI3Kc2β, and PI3Kc2γ; Class III is found mostly in eukaryotes.^[5]^ AKT is a serine/threonine protein kinase with 3 isoforms: AKT1, AKT2, and AKT3, which can respond to upstream PI3K activation, with AKT3 mainly expressed in brain tissue.^[6]^ Recent studies have found that the PI3K/AKT signaling pathway plays a key role in the occurrence and development of fibrotic diseases. There is a lack of effective treatment drugs for visceral fibrosis, and traditional Chinese medicine (TCM) has shown certain effects and advantages in the treatment of fibrotic diseases.^[7–9]^ In recent years, an increasing number of studies have focused on the modulatory effects of TCM on the PI3K/AKT signaling pathway. These studies have revealed that TCM can manipulate the PI3K/AKT signaling pathway via various mechanisms, thereby offering potential therapeutic strategies for fibrotic diseases. This burgeoning field of research provides novel insights and approaches for the management of fibrotic conditions.

## 2. Relationship between PI3K/AKT signaling pathway and fibrotic diseases

### 2.1. Activation of the PI3K/AKT signaling pathway and the regulatory mechanism of fibrotic diseases

Activation of the PI3K/AKT signaling pathway is considered to be an important mechanism in the occurrence and development of fibrotic diseases, and PI3K/AKT signaling plays a key role in the initial stage of epithelial cell injury, the coagulation cascade, immune activation, and fibroblast accumulation, which initiates fibrosis.^[10]^ In addition, the activation of PI3K/AKT signaling pathway can further aggravate the degree of fibrosis by regulating cell survival, proliferation, growth, metabolism, angiogenesis, and metastasis.^[11]^ The activity of PI3K/AKT signaling pathway is regulated by a variety of factors, including intracellular signaling molecules and extracellular environmental factors. (see Tables S1–S4, Supplemental Digital Content, https://links.lww.com/MD/P237, which illustrates the specific information on all single herbs, extracts and TCMs formula).

#### 2.1.1. Myocardial fibrosis

Diffuse myocardial fibrosis results from excessive deposition of collagen fibers throughout the myocardium and is encountered in many chronic heart diseases. This lesion is caused by an altered regulation of fibrocollagen turnover by fibroblasts, promoting excessive deposition of type I and III collagen fibers within the myocardial interstitium and around the intracardiac vessels. Existing evidence suggests that, in addition to the extent of fiber deposition, collagen composition and physicochemical properties of fibers are also associated with the adverse effects of diffuse myocardial fibrosis on cardiac function and clinical outcomes in patients with heart failure.^[12]^ Ischemic cell death during myocardial infarction results in a heterogeneous repair response in which damaged tissue is replaced by fibrotic scars produced by fibroblasts and myofibroblasts. This also causes geometric, biomechanical, and biochemical changes in the uninjured ventricular wall that initiate reactive remodeling processes, including interstitial and perivascular fibrosis. Although initial reparative fibrosis is essential to prevent ventricular wall rupture, an excessive fibrotic response and reactive fibrosis outside the injured area are detrimental because they lead to progressive impairment of cardiac function and ultimately heart failure. Since TGF-β plays a central role in promoting fibroblast proliferation, myofibroblast transdifferentiation, collagen deposition, and myofibroblast survival, inhibition of TGF-β signaling is a promising approach to suppress fibrosis, and enhancing brain natriuretic peptide-mediated cardiac effects would be an attractive strategy to suppress cardiac fibrosis. An alternative approach to enhancing anti-fibrotic signaling by activating cgMP-mediated pathways is inhibition of the cgMP-degrading enzyme phosphodiesterase.^[13]^ The PI3K/AKT pathway transduces most of the metabolic effects of insulin. In addition to cytosolic targets, insulin-stimulated phosphorylated AKT also translocalizes to mitochondria in the myocardium, and activation of mitochondrial AKT1 protects against diabetic cardiomyopathy and improves metabolism outside the heart.^[14]^ Cardiomyocyte dysfunction induced by phenylephrine can be alleviated by regulating the reactive oxygen species (ROS)-induced PI3K/AKT-dependent signaling pathway.^[15]^ Activation of PI3K/AKT/nuclear factor erythroid derived 2-like 2 (Nrf2) signaling pathway can reduce oxidative stress in myocardial tissue of diabetic rats, thereby improving myocardial injury in diabetic rats.^[16]^ Activation of PI3K/AKT/mechanistic target of rapamycin kinase (mTOR) pathway can inhibit excessive autophagy to alleviate myocardial fibrosis induced by myocardial infarction.^[17]^

#### 2.1.2. Pulmonary fibrosis

Idiopathic pulmonary fibrosis (IPF) is a chronic progressive fibrotic interstitial lung disease with unknown etiology. It is identified as one of the most common and severe forms of idiopathic interstitial pneumonia. A large number of studies have revealed the key role of epithelial cell damage in the pathogenesis of pulmonary fibrosis, and some evidence supports the view that alveolar epithelial damage is the core of the disease.^[18]^ TGFβ1-induced epithelial-mesenchymal transition (EMT) of alveolar epithelial cells (AEC) leads to myofibroblast formation and progressive pulmonary fibrosis.^[19]^ It is common to observe abnormal epithelial cells, such as bronchial epithelial cells and proliferative type II AECs, lining areas of honeycomb fibrosis in IPF lung biopsies, and studies have shown that AECs damage is sufficient to cause pulmonary fibrosis.^[20]^ In IPF lung biopsies, significant AECs apoptosis was also found in areas positive for remodeling and areas with high myofibroblast activity, suggesting that AECs apoptosis is involved in the pathogenesis of IPF.^[21]^ PI3K/AKT signaling pathway activates EMT expression^[22]^ and inhibits cell apoptosis.^[23]^ PI3K/AKT signaling pathway is the downstream of TGFβ1, an important signaling molecule in fibrotic diseases, indicating that PI3K/AKT signaling pathway may participate in the process of pulmonary fibrosis through the above pathways.

#### 2.1.3. Hepatic fibrosis

The occurrence, development, and regression of hepatic fibrosis are affected by various cytokines, chemokines, damage-related molecular patterns, and signaling pathways. A large number of studies in recent years have shown that the progression of hepatic fibrosis is closely related to programmed cell death processes such as apoptosis, autophagy, pyroptosis, necroptosis, ferroptosis, brass apoptosis, and PAN apoptosis. Inducing the death of hepatic stellate cells (HSCs) or preventing the death of other hepatocytes can delay or even reverse hepatic fibrosis.^[24]^ TGF-β1 is a key member of the TGF-β superfamily and plays a key role in the development of hepatic fibrosis. Its expression is consistently elevated in affected organs, which is associated with increased ECM deposition. SMAD proteins have been extensively studied as key intracellular effectors of TGF-β1, acting as transcription factors. In the case of hepatic fibrosis, SMAD3 and SMAD4 are pro-fibrotic, whereas SMAD2 and SMAD7 are protective. Deletion of SMAD3 inhibits type I collagen expression and blocks epithelial-myofibroblast transition. In contrast, SMAD2 disruption upregulated type I collagen expression. SMAD4 plays an important role in fibrotic diseases by enhancing SMAD3 reactive promoter activity, while SMAD7 negatively mediates SMAD3-induced fibrosis.^[25]^ In TGF-β1-induced hepatic fibrosis, it inhibits PI3K/AKT/mTOR signaling pathway, inhibits ECM deposition in hepatic fibrosis, affects the changes of lipid metabolism metabolites, regulates choline metabolism, and thus inhibits fibrosis.^[26]^ Inhibition of PI3K/AKT/mTOR signaling pathway can inhibit the proliferation of HSCs, reduce pro-fibrotic markers, and induce apoptosis and cell cycle arrest.^[27]^ Therefore, PI3K/AKT signaling pathway is one of the important pathways in hepatic fibrosis.

#### 2.1.4. Renal fibrosis

Renal fibrosis (RF) is the deposition of fibrotic matrix and scar formation in response to severe or persistent injury. Although it is involved in the wound healing process, persistent fibrosis impairs tissue structure and organ function, eventually leading to renal failure. Chronic kidney injury promotes various pathological changes, including EMT, endothelial-mesenchymal transition (EndMT), and the activation of fibroblasts and pericytes.^[28]^ The Nrf2 signaling pathway may counteract the development of EMT induced by TGF-β1 and renal interstitial fibrosis (RIF), and the anti-fibrosis effect of Nrf2 is conferred through the inactivation of PI3K/AKT signaling.^[29]^ TGF-β is a major regulator of RF and mediates progressive RF through both classical and nonclassical signaling pathways. In canonical SMAD signaling, SMAD3 is a key mediator of tissue fibrosis and mediates RF through a number of noncoding ribonucleic acid.^[30]^ AKT/glycogen synthase kinase 3β (GSK-3β)-mediated Nrf2 activation can reduce ferroptosis at the early stage of folate-induced renal injury, thereby delaying fibrosis progression.^[31]^ Estrogen receptor α/PI3K/AKT signaling pathway synergistically aggravates RF, inflammatory infiltration, EMT, and ECM deposition.^[32]^ Hypoxia is an important microenvironmental factor for the development of RF. Hypoxia induces the expression of Bmi1 mRNA and protein in human renal tubular epithelial cells. The expression of Bmi1 may be directly regulated by hypoxia-inducible factor-1a, forcing Bmi1 expression to induce EMT. Activation of hypoxia-inducible factor-1a/Twist–Bmi1 signaling in renal epithelial cells is associated with the development of chronic kidney disease (CKD) and may promote fibrosis by promoting EMT to regulate PI3K/AKT/snail signaling.^[33]^ The PI3K/AKT pathway plays an important role in RF by mediating multiple signaling pathways and biological processes.

### 2.2. The PI3K/AKT signaling pathway: a novel target for fibrosis treatment

**Figure 1.**
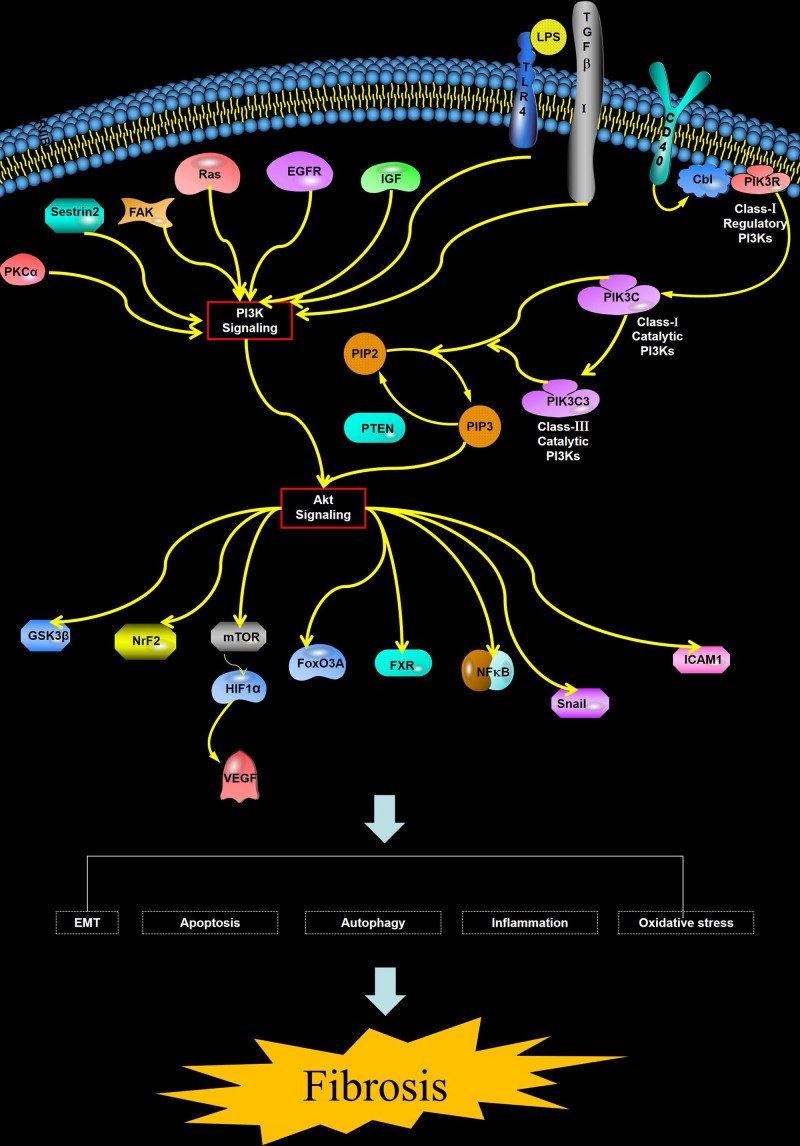
PI3K/AKT and fibrosis. Related pathways and cytokines between the PI3K/AKT pathway and fibrotic diseases. Arrows in the figure indicate the effect of cytokines. AKT = protein kinase B, PI3K = phosphatidylinositol-3-kinase.

Given the pivotal role of the PI3K/AKT signaling pathway in fibrotic diseases, its bidirectional regulation is increasingly recognized as an effective therapeutic strategy for these conditions. Currently, several drugs targeting the PI3K/AKT signaling pathway have been developed and have demonstrated promising anti-fibrotic effects in both experimental and clinical studies. This offers new therapeutic targets and strategies for the management of fibrotic diseases (Fig. 1).

## 3. Advances in TCM intervention on the PI3K/AKT signaling pathway

### 3.1. Impact of individual TCM drugs or extracts on the PI3K/AKT signaling pathway

**Figure 2.**
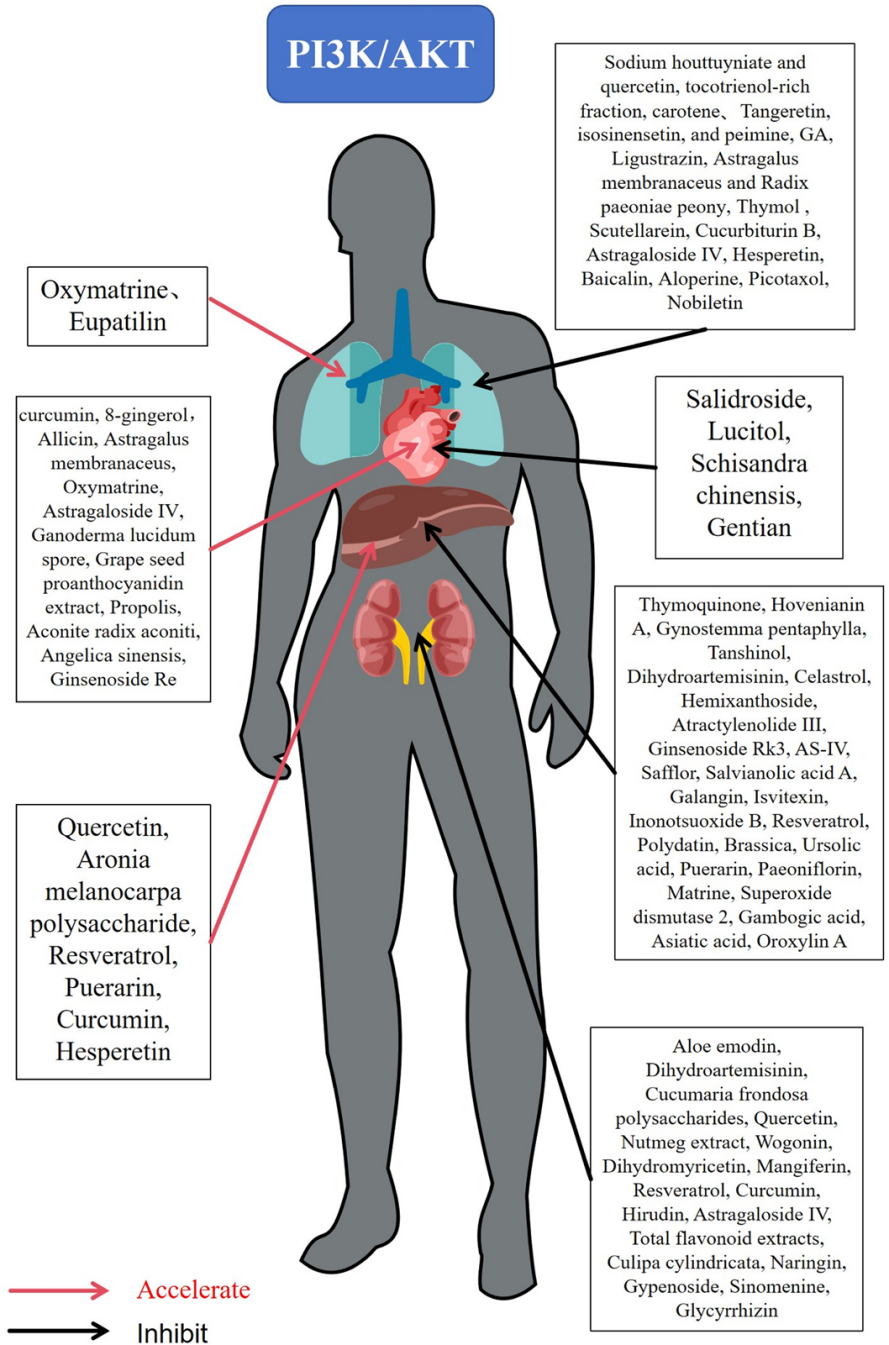
Individual TCM Drugs or Extracts on the PI3K/AKT Signaling Pathway. Red arrows are facilitation and black arrows are inhibition. AKT = protein kinase B, GA = gambogic acid, PI3K = phosphatidylinositol-3-kinase, TCM = traditional Chinese medicine.

In recent years, several individual herbal medicines have been identified to prevent and treat fibrotic diseases by modulating the PI3K/AKT signaling pathway. Certain TCMs can inhibit the onset and progression of fibrosis by suppressing or enhancing the expression or activity of PI3K/AKT. Furthermore, some TCMs can indirectly influence the PI3K/AKT signaling pathway by regulating the signaling molecules upstream or downstream of PI3K/AKT (Fig. 2).

#### 3.1.1. Pulmonary fibrosis

In the study of pulmonary fibrosis, many metabolites have been found to exert their effects by regulating the PI3K/AKT signaling pathway. These metabolites can be broadly divided into the following categories:

[1] Metabolites inhibiting fibrosis by activating the PI3K/AKT signaling pathway: oxymatrine (OMT), an active ingredient in the Chinese herbal medicine Matrine, has an anti-fibrosis effect. OMT attenuated TGF-β1-mediated mitochondrial apoptosis in AEC by activating the PI3K/AKT signaling pathway.^[34]^ Eupatilin is the main metabolite extracted from the traditional herb *Artemisia asiatica* Nakai, which inhibits pulmonary fibrosis by activating Sestrin2/PI3K/AKT/mTOR-dependent autophagy pathway.^[35]^
[2] Metabolites inhibiting fibrosis by inhibiting PI3K/AKT signaling pathway: direct inhibitory effect: Sodium houttuyniate and quercetin can prevent bleomycin (BLM)-induced pulmonary fibrosis in mice by inhibiting AKT and mitogen activated protein kinase (MAPK) pathways.^[36]^ The tocotrienol-rich fraction and carotene inhibit apoptosis and induce TGF-β1/SMAD, PI3K/AKT/mTOR and nuclear factor-kappa B (NF-κB) signaling pathways to improve BLM-induced lung injury and pulmonary fibrosis.^[37]^ Tangeretin, isosinensetin, and peimine may be active metabolites involved in the treatment of pulmonary fibrosis, and have therapeutic effects on IPF by inhibiting epidermal growth factor receptor/PI3K/AKT.^[38]^ Indirect inhibition: GA inhibited high mobility group protein 1 and brahma-related genes 1 (BRG1; SMARCA4) to inhibit PI3K/AKT/mTOR pathway, thereby reducing silicosis pulmonary fibrosis.^[39]^ Ligustrazin blocks paraquat-induced PI3K/AKT/mTOR and hedgehog signaling pathways by increasing the expression of miR-3a, thereby attenuating paraquat-induced pulmonary fibrosis.^[40]^
[3] Metabolites inhibiting fibrosis by regulating PI3K/AKT signaling pathway and other signaling pathways: Astragalus membranaceus and Radix paeoniae peony could alleviate BLM-induced pathological pulmonary fibrosis. Treatment with Astragalus and red paeoniae resulted in decreased mRNA levels of key targets AKT1, HSP90AA1, CASP3, MAPK3, and vascular endothelial growth factor A (VEGFA). In addition, the protein expression levels of AKT1, HSP90AA1 and VEGFA were also decreased. These results support the therapeutic potential of Astragalus and red peony in ameliorating pulmonary fibrosis, mainly in the PI3K-AKT pathway, HIF-1 signaling pathway, and apoptosis.^[41]^ Thymol is a dietary metabolite found in thyme species can up-regulate the expression of miR-29a in the lung and reduce TGF-β and PI3K/AKT signaling, which plays a significant antioxidant and anti-inflammatory role. It can effectively prevent BLM-induced pulmonary fibrosis.^[42]^ Tangeretin can significantly inhibit TGF-β-induced fibroblast activation, and tangeretin can significantly inhibit the activation of PI3K/AKT, Janus kinases (JAK)/ signal transducer and activator of transcription (STAT) and Wnt pathways, thereby inhibiting fibrosis.^[43]^ Scutellarein inhibits the differentiation of fibroblasts into myofibroblasts by inhibiting TGF-β/SMAD signaling, inhibits cell proliferation by inhibiting PI3K/AKT signaling, and increases apoptosis of fibroblasts by affecting BCL-2-associated X (Bax)/B-cell lymphoma-2 (Bcl-2) signaling, thus inhibiting pulmonary fibrosis.^[44]^
[4] Metabolites that inhibit fibrosis by inhibiting the PI3K/AKT signaling pathway and affecting other biological processes.

TGFβ1 can promote PI3K/AKT signaling pathway and play an important role in pulmonary tissue EMT-mediated fibrosis. Cucurbiturin B inhibits pulmonary tissue EMT induced by TGF-β1 by regulating ROS and PI3K/AKT/mTOR pathway.^[45]^ Astragaloside IV (AS-IV) is a natural saponin from Astragalus membranaceus, which shows anti-fibrotic properties in BLM-induced pulmonary fibrosis. AS-IV significantly inhibits TGF-β1/PI3K/AKT-induced Forkhead box O3(FOXO3a) hyperphosphorylation and reverses EMT during fibrosis progression.^[46]^ Hesperetin inhibits EMT by inhibiting PI3K/AKT pathway, thereby reducing BLM-induced pulmonary fibrosis.^[47]^ Diosmetin alleviated TGF-β3-induced EMT by inhibiting ROS generation and inactivating PI3K/AKT and MAPK pathways.^[48]^ Baicalin treatment attenuates fibrosis caused by pulmonary vascular remodeling by inhibiting calpain-1 and PI3K/AKT-mediated endothelial-mesenchymal transition (EndMT).^[49]^ Aloperine is a quinazine alkaloid extracted from Matrine, which has been shown to alleviate oxidative stress, inhibit PI3K/AKT/mTOR signaling and inhibit human platelet-derived growth factor-BB-stimulated cell proliferation in mouse lung fibroblasts and inhibit the differentiation of fibroblasts into myofibroblasts through inhibited TGF-β/SMAD signaling.^[50]^ Picotaxol, a natural hydroxylated analogue of resveratrol, can down-regulate the expression of TGF-β in lung, inhibit PI3K/AKT signaling pathway, reduce ECM deposition, and thus alleviate pulmonary fibrosis.^[51]^ Baicalin can reduce oxidative stress by inhibiting PI3K/AKT and calcium/calmodulin-dependent protein kinase II signaling pathways, thereby alleviating pulmonary fibrosis.^[52]^ Nobiletin is a citrus flavonoid. NOB inhibits amiodarone-induced pulmonary fibrosis by inhibiting the TGF-β3-related PI3K/AKT/mTOR cascade, inhibiting alpha smooth muscle actin (α-SMA) expression and hindering collagen deposition.^[53]^

Pulmonary fibrosis is a debilitating lung disease characterized by excessive scarring and stiffening of lung tissue, resulting in compromised lung function. Central to this process are the TGF-β1 and PI3K/AKT signaling pathways. TGF-β1 can instigate EMT, a key contributor to lung fibrosis. The PI3K/AKT signaling pathway, on the other hand, modulates cell survival and proliferation, and influences cell apoptosis and autophagy. Current research suggests that certain drugs and natural metabolites can impede the progression of pulmonary fibrosis by modulating the TGF-β1 and PI3K/AKT signaling pathways. These metabolites primarily inhibit TGF-β1-induced EMT, activate or suppress the PI3K/AKT signaling pathway, mitigate oxidative stress, inhibit cell proliferation and differentiation, and enhance apoptosis. They also regulate other related signaling pathways (such as HIF-1, MAPK, mTOR, JAK/STAT, Wnt, Nrf1/HO-3, etc) to exert their anti-fibrotic effects. Collectively, these findings provide a compelling basis for the development of innovative therapeutic strategies for pulmonary fibrosis.

#### 3.1.2. Myocardial fibrosis

In the study of myocardial fibrosis, many metabolites have been found to exert their effects by regulating the PI3K/AKT signaling pathway. These metabolites can be broadly divided into the following categories:

(1) Metabolites inhibiting fibrosis by activating the PI3K/AKT signaling pathway: curcumin alleviates oxidative stress and inhibits apoptosis in diabetic cardiomyopathy by activating the Sirt1-Foxo1 and PI3K-AKT signaling pathways.^[54]^ 8-Gingerol (8-Gin) is a series of phenolic substances extracted from ginger. 8-Gin can significantly increase the expression of PI3K/AKT/mTOR signaling pathway related proteins, inhibit ROS generation, cell apoptosis and autophagy by regulating PI3K/AKT/mTOR signaling pathway. It has a cardioprotective effect on ISO-induced myocardial fibrosis.^[55]^ Allicin pretreatment can prevent myocardial ischemia-reperfusion injury and activate the miR-19a-3p/PI3K/AKT pathway, thereby reducing myocardial fibrosis.^[56]^ Astragalus membranaceus (Cal), the main active ingredient of Astragalus membranaceus, has been reported to have therapeutic effects on cardiac dysfunction after myocardial infarction. Cal inhibits inflammation and fibrosis by activating the PI3K-AKT pathway in fibroblasts and heart failure in rats after acute myocardial infarction.^[57]^ OMT preconditioning protects H9c2 cardiomyocytes from H/R injury by activating PI3K/AKT pathway.^[58]^ AS-IV, the main active metabolite of Astragalus membranaceus, can improve cardiomyocyte hypertrophy, apoptosis and fibrosis, and may reduce adriamycin-induced cardiac injury by activating PI3K/AKT pathway.^[59]^ Ganoderma lucidum spore treatment can alleviate STZ-induced fibrosis and myocardial dysfunction by stimulating the PI3K/AKT/mTOR signaling pathway and reducing hyperglycemia, oxidative stress, inflammation, and apoptosis, thus alleviating diabetic cardiomyopathy.^[60]^ Grape seed proanthocyanidin extract can improve cardiac remodeling after myocardial infarction in mice by promoting PI3K/AKT pathway.^[61]^ Propolis is a traditional medicine that has been widely used as an anti-inflammatory and antioxidant for a thousand years. The flavonoid moiety, the main active metabolite of propolis, has a wide range of biological activities and can reduce myocardial fibrosis by promoting the PI3K/AKT pathway.^[62]^ The combination of Aconite radix aconiti and Angelica sinensis can reduce myocardial fibrosis and inflammatory reaction, protect ischemic cardiomyocytes, and reduce myocardial injury. The mechanism may be related to the regulation of PI3K/AKT pathway.^[63]^
(2) Metabolites inhibiting fibrosis by inhibiting PI3K/AKT signaling pathway: Salidroside attenuated myocardial remodeling in DOCA salt-induced mice by inhibiting endothelin-1 and PI3K/AKT/NFκB signaling pathways.^[64]^ Lucitol, a natural triterpenoid metabolite with anti-inflammatory and antiapoptotic activities, has a potential protective effect against cardiovascular disease and prevents cardiac hypertrophy through an anti-inflammatory mechanism, which is due to the inhibition of TLR4-PI3K-AKT-NF-κB signaling.^[65]^
(3) Metabolites that inhibit fibrosis by regulating the PI3K/AKT signaling pathway, as well as other signaling pathways: Schisandra chinensis can effectively improve the heart coefficient, reduce the area of myocardial fibrosis, reduce the serum levels of IL-1β and TNF-α, reduce myocardial cell apoptosis, increase the expression of Bcl-2, and reduce the expression of p-PI3K/PI3K, p-AKT/AKT, P65 and Bax in heart tissue of rats with heart failure.^[66]^ Ginsenoside Re (Gin-Re) treatment promoted AMPKα phosphorylation, decreased TGF-β1 expression, and attenuated SMAD2/3 activation. After Gin-Re treatment, the phosphorylation of FAK, PI3K p110α and AKT was enhanced in the myocardial infarction group. Gin-Re may improve myocardial infarct-induced cardiac dysfunction and attenuate ventricular remodeling by regulating AMPK/TGF-β1/SMAD2/3 and FAK/PI3K p110α/AKT signaling pathways.^[67]^
(4) Metabolites that inhibit fibrosis by inhibiting PI3K/AKT signaling and affecting other biological processes: Gentian has cardioprotective effect by inhibiting PI3K/AKT/FOXO1/3a pathway and activating Notch signaling pathway. It can alleviate myocardial fibrosis, inhibit hypertrophy, reduce autophagy and block EMT by regulating PI3K/AKT/FOXO1/3a and Notch signaling pathway.^[68]^

Myocardial fibrosis, a prevalent cardiac disease, is characterized by the substitution of cardiomyocytes with fibrous tissue, leading to a decline in cardiac function. Central to this process is the PI3K/AKT signaling pathway, which modulates cell survival and proliferation, and influences cell apoptosis and autophagy. Certain drugs and natural metabolites can impede the progression of myocardial fibrosis by modulating the PI3K/AKT signaling pathway. These metabolites primarily exert their anti-fibrotic effects by activating or suppressing the PI3K/AKT signaling pathway, mitigating oxidative stress, inhibiting cell proliferation and differentiation, enhancing cell apoptosis, and regulating other related signaling pathways (such as NFκB, Sirt1-Foxo1, TLR4-NF-κB, AMPK, mTOR, etc). Collectively, these findings provide a compelling theoretical foundation for the development of novel therapeutic strategies for myocardial fibrosis.

#### 3.1.3. Hepatic fibrosis

In the study of hepatic fibrosis, many metabolites have been found to function by regulating the PI3K/AKT signaling pathway. These metabolites can be broadly divided into the following categories:

(1) Metabolites inhibiting fibrosis by activating the PI3K/AKT signaling pathway: quercetin reduces liver injury by inhibiting TGF-β1/SMADs signaling and inhibiting autophagy in BDL-or CCl by activating PI3K/AKT signaling 4-inducing hepatic fibrosis. Quercetin attenuates HSC activation and reduces autophagy by regulating the crosstalk between TGF-β1/SMADs and PI3K/AKT pathways, thereby preventing hepatic fibrosis.^[69]^ Aronia melanocarpa polysaccharide treatment blocked TGF-β1/SMADs pathway to inhibit ECM production and alleviate hepatic fibrosis. In addition, Aronia melanocarpa polysaccharide treatment enhanced the phosphorylation of PI3K/AKT and reduced the expression of its downstream apoptosis-related proteins in the liver, thereby effectively alleviating TAA-induced hepatic fibrosis.^[70]^ Resveratrol (RSV) induces autophagy and activates miR-20a-mediated tension homology deleted on chromosome ten (PTEN)/PI3K/AKT signaling pathway to attenuate hepatic fibrosis.^[71]^ Puerarin attenuates poly (ADP-ribose) polymerase-1 (PARP-1) expression and activates PI3K/AKT pathway to promote mitochondrial homeostasis to prevent nonalcoholic fatty liver disease and hepatic fibrosis induced by high-fat and high-sucrose diet.^[72]^ Curcumin can inhibit autophagy by activating the PI3K/AKT/mTOR signaling pathway, thereby inhibiting the activity of HSCs and promoting apoptosis to inhibit hepatic fibrosis.^[73]^
(2) Metabolites that inhibit fibrosis by inhibiting the PI3K/AKT signaling pathway: Metabolites inhibit the activation of HSCs or the synthesis of ECM by inhibiting the PI3K/AKT signaling pathway, thereby inhibiting hepatic fibrosis. Thymoquinone (TQ), the main active metabolite extracted from medicinal blackgrass, partially alleviates hepatic fibrosis by blocking TLR4 expression and PI3K/AKT phosphorylation on activated HSCs.^[74]^ Hovenianin A alleviates hepatic fibrosis by inhibiting the PI3K/AKT pathway induced by TGF-β3 in HSCs.^[75]^ Gynostemma pentaphylla inhibits the PI3K/AKT pathway to treat hepatic fibrosis.^[76]^ Tanshinol is a water-soluble bioactive monomer purified from the dried root of Salvia miltiorrhiza and has been reported to exert hepatoprotective efficacy in rodents. Tanshinol exerts anti-hepatic fibrosis effect by inhibiting the PI3K/AKT/mTOR/p70S6K1 signaling pathway.^[77]^ Dihydroartemisinin (DHA) induces apoptosis of HSCs by inhibiting PI3K/AKT, thereby inhibiting hepatic fibrosis.^[78]^ Celastrol significantly alleviated autoimmune hepatitis and hepatic fibrosis by inhibiting PI3K/AKT signaling pathway.^[79]^ Hemixanthoside (WO), a flavonoid extracted from Phellosides chinensis, can inhibit the PI3K/AKT/mTOR/NF-κB signaling pathway, thereby inhibiting hepatic fibrosis.^[80]^ Atractylenolide III (ATL III) is a natural metabolite isolated from the plant Atractylodes macrocephala, which affects the PI3K/AKT pathway by inhibiting the phosphorylation of PI3K and AKT, thereby improving hepatic fibrosis induced by bile duct ligation.^[81]^ Ginsenoside Rk3 is found to significantly inhibit PI3K/AKT signaling pathway to improve hepatic fibrosis.^[82]^ AS-IV can inhibit hepatic fibrosis by inhibiting PI3K/AKT/mTOR signaling pathway.^[83]^ Safflor treatment prevented carbon tetrachloride-induced upregulation of HIF-1α, VEGFA, AKT and PI3K, suggesting that safflor may regulate AKT/HIF-1α/VEGF and alleviate hepatic fibrosis.^[84]^
(3) Metabolites that inhibit fibrosis by regulating the PI3K/AKT signaling pathway, as well as other signaling pathways: Hesperetin upregulates antioxidant levels (SOD/GPx/GR/GCLC/HO-1) by triggering the PI3K/AKT-Nrf2 pathway to reduce ROS overproduction and hepatotoxicity. Hesperetin activates the PI3K/AKT-Nrf2 pathway in the liver to increase antioxidant expression. Inhibition of NF-κB activation and secretion of inflammatory cytokines alleviated hepatic steatosis, oxidative stress, inflammatory cell infiltration and fibrosis.^[85]^ Curcumin inhibits the level of ROS and oxidative stress in hepatocytes by activating PPAR-α, and regulates the upstream signaling pathways of AMPK and PI3K/AKT/mTOR, resulting in increased autophagic flux in hepatocytes. In this study, we demonstrated that curcumin effectively reduced the occurrence of EMT and inhibited the production of ECM in hepatocytes by activating autophagy, which provides a potential new therapeutic strategy for hepatic fibrosis.^[86]^ Salvianolic acid A (SA-A), a TCM extracted from the plant Salvia miltiorrhiza, inhibits the PI3K/AKT/mTOR signaling cascade, prevents the stimulation of HSCs, ECM synthesis, and regulates the Bcl-2/Bax and caspase-3 signaling pathways to prevent hepatocyte apoptosis.^[87]^ Galangin inhibits PI3K/AKT and Wnt/β-catenin signaling pathways and increases the ratio of Bax/Bcl-2 to play an anti-hepatic fibrosis role.^[88]^ Isvitexin inhibits hepatic fibrosis by inhibiting PTEN-PI3K-AKT-mTOR axis and GSH metabolic pathway.^[89]^ Inonotsuoxide B is a tetracyclic triterpenoid that can be extracted from Inonotus obliquus. The proliferation and activation of HSC were inhibited by inhibiting PI3K/AKT and extracellular signal-regulated kinase (ERK1/2) signaling pathways.^[90]^ Resveratrol inhibits hepatic fibrosis by blocking NF-κB activation and PI3K/AKT phosphorylation.^[91]^ Polydatin is a metabolite derived from Polygonum cuspidatum, which inhibits the PI3K/AKT/mTOR signaling pathway and increases the expression and activity of transcription factor EB (TFEB), thereby inhibiting hepatic fibrosis.^[92]^ Brassica could inhibit hepatic fibrosis by inhibiting TGF-β1/SMAD, NF-κB and AKT signaling pathways.^[93]^ Ursolic acid (UA) inhibits ERK, PI38K/AKT and p6 MAPK signaling pathways in HSCs, and plays an anti-hepatic fibrosis role.^[94]^ Puerarin plays a protective role in liver tissue by regulating the expression of PPAR-γ and blocking the PI3K/AKT pathway to inhibit the excessive deposition of collagen.^[95]^ Paeoniflorin can inhibit the canonical TGF-β/SMAD signaling pathway and the noncanonical TGF-β/PI3K-AKT signaling pathway to alleviate hepatic fibrosis.^[96]^ Matrine significantly decreased the expression of PI3K and p-AKT and increased the level of PTEN, thereby inhibiting the proliferation, activation and migration of human HSCs.^[97]^ Superoxide dismutase 2 (SOD2) alleviates ROS accumulation and inhibits PI3K/AKT signaling, leading to reduced production of transforming growth factor-β (TGF-β) and nuclear translocation of transcription factor SMAD, thereby inhibiting hepatic fibrosis.^[98]^
(4) Metabolites inhibiting fibrosis by inhibiting PI3K/AKT signaling pathway and affecting other biological processes: gambogic acid (GA), the main metabolite of Garcinia gambogic, inhibits hepatic fibrosis by inhibiting HSP90, inhibiting PI3K/AKT pathway, inducing autophagy and mitochondria-mediated apoptosis.^[99]^ Asiatic acid (AA) is a triterpenoid metabolite isolated from Centella asiatica. AA has effective anti-inflammatory and antioxidant activities, and regulates PI3K/AKT/mTOR signaling pathway to inhibit the activation of HSCs and ECM synthesis, thereby inhibiting hepatic fibrosis.^[100]^ Oroxylin A, an extract of scutellaria baicalensis, inhibits PI3K/AKT/mTOR by scavenging ROS to activate autophagy, thereby inhibiting hepatic fibrosis.^[101]^

Hepatic fibrosis, a prevalent liver disease, is characterized by the substitution of hepatocytes with fibrous tissue, leading to a decline in liver function. Central to this process is the PI3K/AKT signaling pathway, which modulates cell survival and proliferation, and influences cell apoptosis and autophagy. Certain TCMs and natural metabolites can impede the progression of hepatic fibrosis by modulating the PI3K/AKT signaling pathway. These metabolites primarily exert their anti-fibrotic effects by activating or suppressing the PI3K/AKT signaling pathway, mitigating oxidative stress, inhibiting cell proliferation and differentiation, enhancing apoptosis, and regulating other related signaling pathways (such as TGF-β1/SMADs, NF-κB, mTOR, ERK1/2, PPAR-α, AMPK, etc). Collectively, these findings provide a compelling theoretical foundation for the development of novel therapeutic strategies for hepatic fibrosis.

#### 3.1.4. Renal fibrosis

In the study of RF, many metabolites have been found to exert their effects by regulating the PI3K/AKT signaling pathway. These metabolites can be broadly divided into the following categories:

(1) Metabolites inhibiting fibrosis by inhibiting PI3K/AKT signaling pathway: Aloe emodin ameliorated TGFβ1-induced RF by inhibiting PI3K/AKT/mTOR signaling pathway.^[102]^ DHA can alleviate RF by regulating the proliferation and differentiation of fibroblasts by reducing the PI3K/AKT pathway.^[103]^ Cucumaria frondosa polysaccharides can inhibit PI3K/AKT/NF-κB signaling pathway and improve TGF-β1-induced inflammation and RIF in HK-2 cells.^[104]^ Quercetin can minimize RF and apoptosis in rats with chronic renal failure by inhibiting the PI3K/AKT pathway.^[105]^ Nutmeg extract has beneficial effects on diabetic nephropathy (DN), and this mechanism has been confirmed to inhibit Ras/PI3K/AKT and RF-related proteins.^[106]^ Wogonin alleviates renal tubular epithelial injury in DN by inhibiting PI3K/AKT/NF-κB signaling pathway.^[107]^
(2) Metabolites that inhibit fibrosis by regulating the PI3K/AKT signaling pathway, as well as other signaling pathways: Hypericin (one of the active metabolites of Kushen) could partially restore TGF-β1-induced EMT, PI3K/AKT pathway activation and intercellular cell adhesion molecule-1 (ICAM1) upregulation. However, these effects of hypericin could be reversed by ICAM1 overexpression, which inhibited RF through the PI3K/AKT/ICAM1 axis.^[108]^ Dihydromyricetin has a variety of pharmacological activities. PTEN is the target gene of miR-155-5p. Dihydromyricetin promotes autophagy and reduces RF by inhibiting miR-155-5p/PTEN signaling pathway and PI3K/AKT/mTOR signaling pathway.^[109]^ Mangiferin down-regulated TGF-β1, upregulated PTEN, and decreased the phosphorylation of PI3K and AKT. These findings suggest that mangiferin can reduce the TGF-β1-mediated elevation of Col I, FN, and α-SMA through the PTEN/PI3K/AKT pathway, thereby reducing inflammation and oxidative stress in DN, thereby inhibiting RIF.^[110]^ Resveratrol inhibits the myofibroblast phenotype and fibrosis formation in UUO kidneys by targeting fibroblast-myofibroblast-myofibroblast differentiation (FMD) and EMT. The anti-fibrotic effect of resveratrol was associated with the decreased proliferation of renal tubular epithelial cells in the interstitium and renal tubules, resulting in the inhibition of the activity of proliferation-related signaling pathways, including MAPK, PI3K/AKT, Wnt/β-catenin and JAK2/STAT3 pathways.^[111]^
(3) Metabolites inhibiting fibrosis by inhibiting the PI3K/AKT signaling pathway and affecting other biological processes: Curcumin inhibits EMT and inflammatory responses by inhibiting TLR4/NF-κB and PI3K/AKT pathways, thereby inhibiting RIF.^[112]^ PI3K/AKT pathway is an important pathway of AS-IV. AS-IV inhibits AKT phosphorylation, blocks GSK-3β phosphorylation, restores GSK-3β activity, facilitates β-catenin degradation, and thus prevents EMT. AS-IV alleviates RF through AKT1/GSK-3β pathway.^[113]^ Total flavonoid extracts (PTFS) inhibited TGF-β1-induced EMT progression by blocking miR-21/PTEN/PI3K/AKT signaling pathway.^[114]^ Culipa cylindricata, a medicinal plant used in TCM, has been used to treat CKD by inhibiting the PI3K/AKT pathway by down-regulating liver X receptor α, thereby attenuating M2 macrophage polarization and thereby alleviating RF.^[115]^ Curcumin inhibits the PI3K/AKT/mTOR pathway, and curcumin is a promising therapeutic agent for RF. Its anti-fibrosis effect may be mediated by inhibiting NOD-like receptor thermal protein domain associated protein 3 inflammasome activity by regulating autophagy and protecting mitochondrial function in rats.^[116]^ Naringin (NRG) can improve renal dysfunction by inhibiting PI3K/AKT signaling pathway, reduce the release of inflammatory cytokines, and reduce the expression of α-SMA and type I collagen to alleviate RF.^[117]^ Gypenoside (GP) can inhibit PI3K/AKT signaling by up-regulating miR-378a-5p in NRK-49F cells stimulated by TGF-β1, thereby reducing the ECM metabolites secreted by NRK-49F cells to alleviate fibrosis.^[118]^ Sinomenine can improve the progression of RF by affecting the expression of miR-204-5p and inhibiting the PI3K-AKT pathway to activate autophagy in BMSC-exo.^[119]^ Glycyrrhizin activates autophagy by inhibiting the PI3K/AKT/mTOR pathway, thereby inhibiting TGF-β1-induced fibrosis.^[120]^

RF, a prevalent kidney disease, is characterized by the substitution of renal cells with fibrous tissue, leading to a decline in renal function. Central to this process is the PI3K/AKT signaling pathway, which modulates cell survival and proliferation, and influences cell apoptosis and autophagy. Certain drugs and natural metabolites can impede the progression of RF by modulating the PI3K/AKT signaling pathway. These metabolites primarily exert their anti-fibrotic effects by suppressing the PI3K/AKT signaling pathway, mitigating oxidative stress, inhibiting cell proliferation and differentiation, enhancing apoptosis, and regulating other related signaling pathways (such as TGF-β1, NF-κB, mTOR, PTEN, etc). Collectively, these findings provide a compelling theoretical foundation for the development of novel therapeutic strategies for RF.

### 3.2. Effect of traditional Chinese medicine metabolite on PI3K/AKT signaling pathway

**Figure 3.**
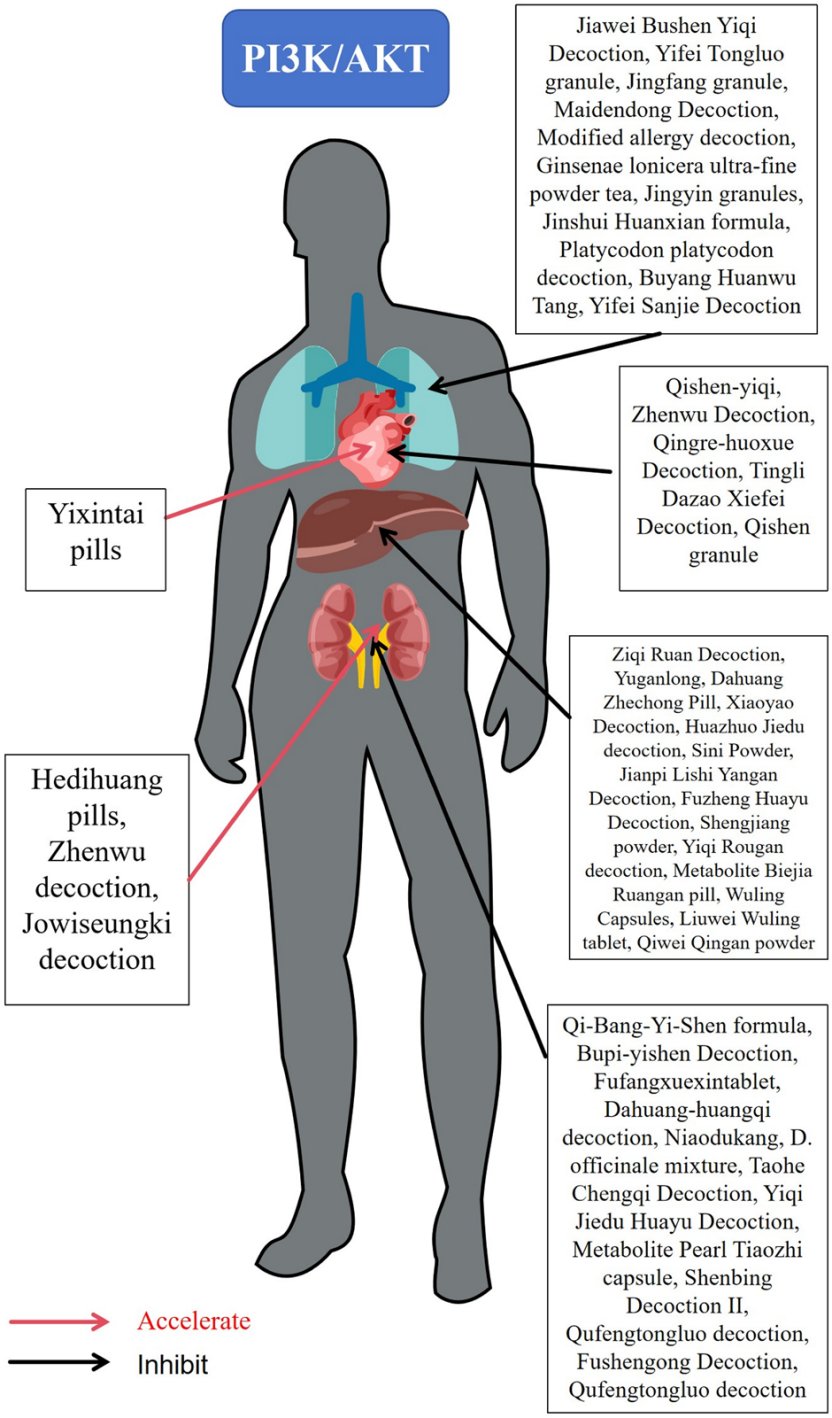
Traditional Chinese medicine metabolite on PI3K/AKT signaling pathway. Red arrows are facilitation and black arrows are inhibition. AKT = protein kinase B, PI3K = phosphatidylinositol-3-kinase.

TCM metabolite is an important means of TCM treatment, which can exert a stronger effect on PI3K/AKT signaling pathway through the synergistic effect of multiple drugs. Some studies have found that some TCM metabolites can regulate multiple links of the PI3K/AKT signaling pathway at the same time, thereby more effectively preventing and treating fibrotic diseases (Fig. 3).

#### 3.2.1. Pulmonary fibrosis

Jiawei Bushen Yiqi decoction reversed BLM-induced leukocyte level, pulmonary inflammatory lesions and fibrosis collagen deposition in mice by inhibiting the PI3K/AKT pathway, and reduced the levels of α-SMA, Col1age1 and TGF-β.^[121]^ Yifei Tongluo granule can treat pulmonary fibrosis by inhibiting EMT, fibroblast proliferation, fibroblast to myofibroblast transformation, myofibroblast anti-apoptosis, collagen expression and other mechanisms through PI3K/AKT pathway and MAPK pathway.^[122]^ Jingfang granule has obvious protective effect on BLM-induced acute lung injury by down-regulating the PI3K/AKT/mTOR signaling pathway, thereby regulating glycolysis/glycogenesis and pyruvate metabolism.^[123]^ Maidendong decoction inhibits pulmonary fibrosis by regulating M3 macrophage polarization through inhibiting PI3K/AKT/FOXO3a signaling pathway mediated fibroblast activation.^[124]^ Modified allergy decoction can reduce inflammation and pulmonary fibrosis by inhibiting PI3K/AKT to improve PM2.5-induced lung injury.^[125]^ The active ingredients of Ginsenae lonicera ultra-fine powder tea have been shown to have anti-inflammatory and anti-oxidative effects and can improve pulmonary fibrosis by inhibiting the PI3K/AKT pathway.^[126]^ Jingyin granules can inhibit JAK2/STAT3, NF-κB, PI3K-AKT, TNF and ERK1/2 signaling pathways to mediate pulmonary fibrosis.^[127]^ Jinshui Huanxian formula is a TCM that has been shown to reduce IPF and improve pulmonary fibrosis by inhibiting the epidermal growth factor receptor/PI3K/AKT signaling pathway to inhibit fibroblast activation.^[38]^ Platycodon platycodon decoction inhibits cell apoptosis by regulating the expression of apoptosis-related proteins Bax, Caspase8 and Caspase9 through PI3K/AKT signaling pathway, thus preventing pulmonary fibrosis.^[128]^ Yangqing Chenfei formula inhibited the activation of PI3K/AKT, JAK/STAT and Wnt pathways to inhibit pulmonary fibrosis.^[43]^ Buyang Huanwu Tang inhibits epithelial-to-mesenchymal transition of cells by inhibiting TGF-β3 activation of PI1K/AKT signaling pathway, thereby alleviating pulmonary fibrosis.^[129]^ Yifei Sanjie decoction can induce autophagy by inhibiting the phosphorylation of PI3K-AKT-mTOR pathway, thereby down-regulating the expression of collagen in lung fibroblasts.^[130]^

#### 3.2.2. Myocardial fibrosis

Qishen-yiqi dropping pills may have anti-myocardial fibrosis effect through HIF-1 signaling pathway, forkhead box O subfamily protein (FoxO) signaling pathway, TNF signaling pathway, PI3K-AKT signaling pathway and other enriched pathways, and it reduces the expression of myocardial p62. The ratio of p-PI3K/PI3K, p-AKT/AKT and p-mTOR/mTOR in myocardium was decreased. This recipe affects anti-reactive myocardial fibrosis in a dose-dependent mechanism mediated by activation of myocardial autophagy by the PI3K/AKT/mTOR pathway.^[131–133]^ Zhenwu decoction improved cardiac function, myocardial pathology and myocardial fibrosis through TGFβ/SMAD-3 signaling pathway. In vivo experiments showed that Zhenwu decoction could improve oxidative stress response and myocardial cell apoptosis through PI3K/AKT signaling pathway, and reduce the levels of inflammatory factors and the protein expression of NF-κB p65, IL-6 and TNF-α.^[134]^ Rhodiola granule can improve myocardial fibrosis by regulating HIF-1/VEGF/PI3K-AKT signaling pathway.^[135]^ Huoxue Wentong decoction can improve myocardial fibrosis through PI3K/AKT pathway.^[136]^ Qingre-Huoxue decoction activates autophagy and inhibits the expression of inflammatory factors by inhibiting PI3K/AKT signaling pathway, which can reduce the degree of myocardial fibrosis, down-regulate serum inflammatory factors, promote autophagy in rats with myocardial infarction, and play a protective role in the myocardium.^[137]^ Tingli Dazao Xiefei decoction can improve cardiac function and prevent cardiac injury by inhibiting the activation of PI3K/AKT and MAPK signaling pathways.^[138]^ Metabolite Danshen dropping pills can improve ischemic myocardial inflammation by synergistically regulating MAPK, PI3K/AKT and peroxisome proliferators-activated receptor (PPAR) signaling pathways.^[139]^ Qishen granule can significantly inhibit the activities of SMAD3 and PI3K-GSK-3β signaling pathways in cardiac fibroblasts.^[140]^ Yixintai pills can inhibit myocardial cell apoptosis and ventricular remodeling in rats by up-regulating PI3K/AKT/GSK-3β signaling, thereby protecting cardiac function.^[141]^

#### 3.2.3. Hepatic fibrosis

Ziqi Ruan decoction has a good anti-hepatic fibrosis effect, and its mechanism is related to anti-oxidative stress, anti-inflammation, inhibition of PI3K/AKT/mTOR signaling pathway, and inhibition of HSC activation.^[142]^ Yuganlong (YGL) is a traditional Chinese herbal formula that inhibits the PI3K/AKT, Ras/ERK and JAK1/STAT3 signaling pathways in carbon tetrachloride to improve hepatic fibrosis.^[143]^ Dahuang Zhechong Pill treatment significantly down-regulated the protein levels of PI3K and phosphorylated AKT, as well as fibrosis markers, to alleviate hepatic fibrosis.^[144]^ Xiaoyao decoction significantly down-regulated the JAK-STAT and PI3K-AKT-FoxO signaling pathways, thereby inhibiting hepatic fibrosis.^[145]^ Huazhuo Jiedu decoction can inhibit TGF-β1/PI3K/AKT to mediate the prevention of hepatic fibrosis.^[146]^ Sini powder inhibits PI3K/AKT/ farnesoid X receptor to improve hepatic fibrosis.^[147]^ Jianpi Lishi Yangan decoction promotes mitophagy by inhibiting the PI3K/AKT/mTOR pathway, thereby improving liver injury and hepatic fibrosis.^[148]^ Fuzheng Huayu decoction prevents hepatic fibrosis by blocking IGF/PI3K pathway and inhibiting HSC activation.^[149]^ FZHY upregulated the expression of PI3K while down-regulating the expression of caspase-3 and BAX/BCL-W in cells treated with TNFα. Conversely, in cells induced by TGFβ, FZHY down-regulated the expression of PI3K and upregulated the expression of caspase-3 and BAX/BCL-W. FZHY inhibited the proliferation of HSCs induced by TGF-β1. It can also attenuate the decrease of HPG activity induced by TNF-α, thereby protecting hepatic parenchymal cells and promoting the apoptosis of HSCs.^[150]^ Shengjiang powder can improve nonalcoholic fatty liver disease and hepatic fibrosis induced by high-fat diet by inhibiting the activation of AKT/mTOR/S6 pathway in rats.^[151]^ Yiqi Rougan decoction has shown long-lasting effects in the treatment of hepatic fibrosis, reducing HSC activity and ECM deposition by inhibiting the PI3K/AKT pathway.^[152]^ Metabolite Biejia Ruangan pill has a cumulative inhibitory effect on PI3K/AKT/NF-κB activation and can alleviate hepatic fibrosis.^[153]^ Wuling capsules can inhibit hepatic fibrosis by regulating PI3K-AKT signaling pathway, reducing the levels of TNF-α and IL-6 inflammatory factors, and inhibiting the excessive deposition of collagen.^[154]^ Liuwei Wuling tablet can alleviate the inflammatory environment and fibrosis of liver cells by inhibiting the PI3K/AKT/NF-κB signaling pathway.^[155]^ Qiwei Qingan powder can induce the apoptosis of HSCs by inhibiting PI3K/AKT, thus alleviating the progression of hepatic fibrosis.^[156]^

#### 3.2.4. Renal fibrosis

Huangqi and Angelica decoction (A&A) is a TCM for treating renal diseases, especially RIF. A&A alleviates RF by regulating cell cycle, limiting cell apoptosis and inhibiting inflammation through PI3K-AKT pathway.^[157]^ Qi-Bang-Yi-Shen formula treatment can significantly reduce the protein expression of PI3K, AKT and ERK1/2 in renal tissue of DKD rats, but increase the level of PPARγ, which can improve renal injury and fibrosis in DKD rats by regulating PI3K/AKT, ERK and PPARγ signaling pathways.^[158]^ Hedihuang pills can up-regulate the expression of insulin-like growth factor 1, promote the binding of insulin-like growth factor 1 to insulin-like growth factor 1 receptor, and then activate PI3K/AKT/mTOR signaling pathway, thereby inhibiting autophagy to reduce RF.^[159]^ Bupi-Yishen decoction reduced PI3K/AKT signaling and RF in a dose-dependent manner.^[160]^ Fufangxuexin tablet inhibited the activation of PI3K/AKT signaling pathway and inhibited the formation of RF.^[161]^ Dahuang-huangqi decoction reduces the mRNA and protein expression of core targets involved in inflammatory pathways, such as PI3K/AKT and TLR4/NF-κB, to treat RF.^[162]^ Niaodukang mixture can inhibit the expression of PDPK1 by up-regulating the expression of mir-129-5p, and then inhibit the PI3K/AKT pathway to improve RF.^[163]^ D. officinale mixture (DMix) is a TCM widely used for the prevention and treatment of DN. DMix inhibited the phosphorylation of PI3K, AKT and mTOR, and increased the protein and mRNA expression levels of microtubule-associated protein 1 light chain 3 and Beclin-1 in renal tissues of DN rats. Therefore, DMix has a protective effect on the kidney of DN rats, which may be related to the inhibition of PI3K/AKT/mTOR signaling pathway and activation of renal autophagy by this traditional drug.^[164]^ Taohe Chengqi decoction may inhibit inflammatory process, reduce ECM deposition and reverse EMT through PI3K/AKT/mTOR and HIF-1α/VEGF signaling pathways, thereby exerting its effect on RF.^[165]^ Zhenwu decoction treatment reduced the expression levels of TGF-β and Wnt, and increased the expression levels of Nrf2, PI3K and AKT in renal tissues. In addition, ZWT reduced cell apoptosis and fibrosis by regulating the expression levels of caspase-3, Bax and α-SMA.^[166]^ Yiqi Jiedu Huayu decoction activates AMPK pathway and inhibits PI3K/AKT and mTOR pathways. Yiqi Jiedu Huayu decoction can further inhibit mTOR pathway and promote autophagy by regulating PI3K/AKT and AMPK pathways, thereby improving podocyte injury, protecting renal function and reducing RF.^[167]^ Metabolite Pearl Tiaozhi capsule can prevent renal injury, inflammation and fibrosis in mice with hyperuricemia-induced nephropathy by promoting uric acid excretion and inhibiting PI3K/AKT/NF-κB signaling pathway.^[168]^ Jowiseungki decoction is a prescription commonly used in traditional medicine clinics to treat diabetic complications or DN. Jowiseungki decoction can improve the symptoms of STZ-induced DN mice by inhibiting renal dysfunction. In particular, it protects the kidney by inhibiting fibrosis and inflammation by regulating protein kinase C alpha/PI3K/AKT and NF-κB/α-SMA signaling pathways.^[169]^ The relationship between Cordyceps ginseng decoction and CKD is related to activating autophagy, promoting mitochondrial degradation and reducing tissue oxidative stress damage, mainly mediated by PI3K/AKT pathway.^[170]^ Shenbing decoction II can not only significantly improve the renal function and fibrosis of rats, but also significantly down-regulate the expression of p53, p-PI3K and p-AKT, thereby alleviating the process of fibrosis.^[171]^ Fushengong decoction can protect renal function and reduce fibrosis by regulating PTEN/PI3K/AKT/NF-κB pathway.^[172]^ Qufengtongluo decoction may inhibit PI3K/AKT signaling pathway by activating PTEN and inhibiting TGF-β to improve RF.^[173]^

Given the significant correlation between organ fibrosis and functional impairment, along with the associated poor prognosis, the primary objectives in the clinical management of fibrotic diseases have shifted towards the maximal control of fibrosis progression and the preservation of remaining organ function. In clinical studies, the efficacy of TCM in the treatment of fibrotic diseases has garnered considerable recognition. Several clinical trials have indicated that TCM interventions can significantly alleviate symptoms and improve the quality of life for patients suffering from fibrotic conditions. In certain cases, TCM has even been shown to reverse the fibrosis process.^[174,175]^ TCM, grounded in the principles of syndrome differentiation and holistic treatment, demonstrates a comprehensive regulatory effect, particularly evident in its efficacy in treating fibrosis. This therapeutic effect may be mediated through the modulation of the PI3K/AKT signaling pathway. TCM metabolites have the capacity to inhibit both the expression and activity of the PI3K/AKT signaling pathway, as well as prevent its translocation to the nucleus. This inhibition subsequently suppresses the regulation of downstream genes. Consequently, this mechanism impedes fibrocyte proliferation and ECM accumulation, thereby hindering the onset and progression of fibrosis. Certain metabolites derived from TCM have the capacity to modulate the expression or activity of the PI3K/AKT signaling pathway, thereby influencing autophagy. The p110α isoform has been shown to enhance the cellular antioxidant defense system through the Nrf2-ARE dependent pathway, effectively preventing the accumulation of ROS and maintaining ROS levels within an optimal range. Simultaneously, p110α can activate the AKT protein, which serves to inhibit autophagy. In contrast, when ROS levels are elevated, p110β can facilitate autophagy by interacting with Rab5, thereby enabling cellular adaptation to increased ROS levels.

Simultaneously, p110β has the capacity to activate FoxO, thereby promoting autophagy. TCM metabolites can also exert indirect effects on the activity of the PI3K/AKt signaling pathway by modulating both upstream and downstream signaling molecules associated with this pathway. Furthermore, TCM metabolites influence the PI3K/AKT signaling pathway through their impact on ECM remodeling. The remodeling of the ECM is a critical characteristic of fibrosis and serves as a significant regulator of the PI3K/AKT signaling pathway. Certain TCMs have the potential to modify the activity of PI3K/AKT by altering the composition and structure of the ECM. For example, specific TCMs can inhibit the excessive accumulation of the ECM, thereby suppressing the activation of PI3K/AKT. This mechanism not only prevents the onset and progression of fibrosis but also contributes to the amelioration of preexisting fibrosis.

## 4. Challenges and prospects of TCM intervention on PI3K/AKT signaling pathway in the treatment of fibrotic diseases

### 4.1. Problems in the study of TCM intervention on PI3K/AKT signaling pathway

While advancements have been achieved in the treatment of fibrotic diseases through TCM interventions targeting the PI3K/AKT signaling pathway, several challenges continue to impede progress. Firstly, the mechanisms underlying TCM’s action are complex and may simultaneously influence multiple facets of the PI3K/AKT signaling pathway, thereby complicating the investigation of its mechanisms. Secondly, the active constituents and specific targets of TCM remain unclear, which limits the scope and effectiveness of its clinical applications. Lastly, the research methodologies and evaluation criteria employed in TCM studies have not yet been fully refined, adversely affecting the precision and reliability of the findings.

### 4.2. Future research directions and prospects of TCM intervention in PI3K/AKT signaling pathway

Despite the challenges previously mentioned, research concerning the modulation of the PI3K/AKT signaling pathway by TCM in the treatment of fibrotic diseases remains highly promising. Firstly, a deeper understanding of the mechanisms underlying TCM’s action may facilitate the identification of novel therapeutic targets, thus offering new strategies for the management of fibrotic diseases. Secondly, the efficacy and safety of treatments can be enhanced through the research and development of TCM that specifically targets the PI3K/AKT signaling pathway. Finally, by refining research methodologies and evaluation criteria, the scientific rigor and reliability of the studies can be improved, thereby advancing the clinical application of TCM in the treatment of fibrotic diseases.

## 5. Conclusions

In the management of fibrotic diseases through TCM and its associated prescriptions, a predominant array of herbal remedies is employed. This suggests that within the framework of TCM, the etiology of fibrosis is primarily attributed to pathological factors including phlegm-dampness, water dampness, and blood stasis. These factors are considered to be the principal contributors to the impairment of organ functions, ultimately precipitating the development of fibrosis. TCM treatment methodologies emphasize the expulsion of pathogenic factors through various approaches, including the promotion of blood circulation, the resolution of stasis, the dispersion of phlegm, and the drainage of dampness. Concurrently, tonifying agents are employed to bolster the body’s righteous qi (is a view in traditional Chinese medicine that represents resistance in the body). The ultimate objective is to achieve a harmonious balance of yin and yang within the body, thereby ensuring the stability of the internal environment.

Significant advancements have been achieved in the treatment of fibrotic diseases through the modulation of the PI3K/AKT signaling pathway via TCM. Specific herbal medicines, including both individual herbs and their metabolites, have been identified as effective in preventing and treating fibrotic diseases by either directly or indirectly influencing the PI3K/AKT signaling pathway. Clinical studies have further validated the efficacy of TCM in the management of fibrotic diseases. Nevertheless, the intricate mechanisms underlying the action of TCM, the uncertainty regarding its active constituents and targets, as well as the necessity for refinement in research methodologies and evaluation criteria, continue to pose significant challenges.

Future research should investigate the specific mechanisms by which TCM intervenes in the PI3K/AKT signaling pathway, as well as identify its active ingredients and targets. This approach aims to enhance both the efficacy and safety of treatment. Concurrently, it is essential to refine research methodologies and evaluation criteria to strengthen the scientific rigor and reliability of the findings. Furthermore, there is a need to increase the volume of clinical research to validate the effectiveness of TCM in the treatment of fibrotic diseases and to facilitate its clinical application. Overall, the intervention of TCM in the PI3K/AKT signaling pathway for the treatment of fibrotic diseases offers significant research opportunities and holds considerable potential for clinical implementation.

Enhancing mechanistic analysis: The application of advanced biotechnological techniques, such as gene editing, proteomics, and metabolomics, facilitates comprehensive investigations into the mechanisms by which TCM and its bioactive components affect fibrosis via the PI3K/AKT signaling pathway and its associated upstream and downstream molecular networks. This research aims to elucidate the specific mechanisms of action of TCM in cell signal transduction, gene expression regulation, and epigenetic modifications, thereby establishing a scientific foundation for targeted therapeutic interventions.

Identification of active ingredients and specific therapeutic targets: This study employs systematic methodologies that encompass the isolation of chemical compositions, structural elucidation, and bioactivity screening to identify the principal bioactive components of TCM that exert direct effects on the PI3K/AKT signaling pathway, along with their corresponding molecular targets. Furthermore, the research utilizes computational simulations and bioinformatics tools to predict potential interactions between drugs and targets, thereby facilitating the expedited development of innovative therapeutic agents.

Collaborative research endeavors: The integration of genomic, transcriptomic, and proteomic analyses, in conjunction with other omics technologies, aims to provide a comprehensive examination of the effects of TCM interventions on gene expression profiles and protein interaction networks associated with fibrosis. This multifaceted approach seeks to elucidate the complex and multidimensional mechanisms underlying the actions of TCM, thereby aiding in the development of comprehensive treatment strategies.

The standardization and globalization of clinical research: This study underscores the critical need for high-quality, large-scale, multi-center randomized controlled clinical trials aimed at standardizing research methodologies, data collection procedures, and analytical techniques. Such efforts are essential for enhancing the reliability and reproducibility of research findings. Additionally, it advocates for the promotion of international collaborations and exchanges to develop universally recognized evaluation standards and guidelines for TCM, thereby facilitating its application and dissemination in the global treatment of fibrotic diseases.

The exploration of personalized medicine involves the customization of treatment strategies based on individual differences and the heterogeneity of diseases among patients. This investigation aims to assess the potential of TCM in the personalized modulation of the PI3K/AKT signaling pathway for the treatment of fibrosis. By employing genotyping and biomarker detection techniques, precision medicine approaches can be utilized to optimize therapeutic outcomes and improve the quality of life for patients.

## Author contributions

**Conceptualization:** Shu-ping Huang.

**Data curation:** Yu Chen.

**Formal analysis:** Min Zhu.

**Funding acquisition:** Wei-hong Li.

**Investigation:** Ze-chao Zhang.

**Methodology:** Chang-jie Shang.

**Resources:** Min Zhu.

**Writing – original draft:** Shu-ping Huang.

**Writing – review & editing:** Shu-ping Huang, Ze-chao Zhang, Wei-hong Li.

## Supplementary Material


